# ERA-CRISPR/Cas12a-based, fast and specific diagnostic detection for *Chlamydia pneumoniae*

**DOI:** 10.3389/fcimb.2024.1477422

**Published:** 2024-11-01

**Authors:** Yanxia Zhou, Zijun Yan, Shi Zhou, Weiwei Li, Hongyu Yang, Hongliang Chen, Zhongliang Deng, Qilin Zeng, Peiyuan Sun, Yimou Wu

**Affiliations:** ^1^ The Institute of Pathogenic Biology, Hengyang Medical School, University of South China, Hengyang, China; ^2^ Department of Clinical Laboratory, The Second People’s Hospital of Foshan, Foshan, China; ^3^ Department of Infection Control, The First People’s Hospital of Chenzhou, Chenzhou, China; ^4^ Department of Public Health Laboratory Sciences, College of Public Health, Hengyang Medical School, University of South China, Hengyang, China

**Keywords:** *Chlamydia pneumoniae*, OmpA gene, CRISPR/Cas12a, ERA, pathogenic bacteria detection, trans-cleavage

## Abstract

*Chlamydia pneumoniae (C. pneumoniae)* is a specialized intracellular parasitic pathogen capable of causing pneumonia, sinusitis, bronchitis, and other respiratory diseases, which pose significant public health challenges. Therefore, rapid, accurate, and sensitive diagnosis is crucial for the prevention and treatment of respiratory diseases caused by *C. pneumoniae*. In this study, we combined enzymatic recombination amplification (ERA) with the clustered regularly interspaced short palindromic repeats (CRISPR)/CRISPR-associated protein (Cas) 12a system (CRISPR/Cas12a) to develop a dual detection platform termed the Cpn-ERA-CRISPR/Cas12a dual system. This system integrates both the ERA-CRISPR/Cas12a fluorescence system and the ERA-CRISPR/Cas12a lateral flow system. Detection results can be measured using a fluorescence detector or observed with the naked eye on lateral flow strips. The fluorescence system and the lateral flow system detect *C. pneumoniae* in 30 minutes and 15 minutes, respectively. This dual system exhibits no cross-reactivity with the other seven pathogens, demonstrating high specificity, and achieves a sensitivity of 10^0^ copies/µL. Additionally, the Cpn-ERA-CRISPR/Cas12a dual system was employed to analyze 39 clinical samples, comprising 19 positive and 20 negative samples. The detection rate for positive samples was 100%, with no positive results in the negative samples, indicating a high level of concordance with qPCR results. In summary, the Cpn-ERA-CRISPR/Cas12a dual system represents a novel tool for diagnosing *C. pneumoniae* and holds promising application potential in grassroots community hospitals.

## Introduction

*C. pneumoniae* is a zoonotic pathogen that can cause respiratory diseases, including pneumonia, bronchitis, and pharyngitis, as well as chronic obstructive pulmonary disease and cardiovascular conditions such as atherosclerosis. Additionally, there is a potential link between *C. pneumoniae* infection and multiple sclerosis and Alzheimer’s disease; however, the evidence supporting this association remains insufficient (Kuo et al., 1995; Sessa et al., 2008; Roulis et al., 2013; Elwell et al., 2016).

At present, numerous clinical methods exist for the identification and diagnosis of *C. pneumoniae* infections. Among these, isolation culture represents the most specific method; however, its clinical utility is limited due to challenges posed by the harsh culture conditions, the organism’s poor survival in cell culture, and the extended time required for this method (Kuo et al., 1995; Hisada et al., 2021). Serological diagnostics, including the complement fixation test (CF), enzyme-linked immunosorbent assay (ELISA), and microimmunofluorescent antibody assay (MIF), are crucial for the laboratory diagnosis of acute *C. pneumoniae* infections (Verkooyen et al., 1998; Hammerschlag, 2003). Although MIF is considered the “gold standard” detection, it has several drawbacks, including cross-reactivity with other pathogens, a lack of standardized testing protocols between laboratories, the necessity for matched serum samples (ideally collected 2-4 weeks apart), and variability in the experience of laboratory personnel in interpreting results (Villegas et al., 2010; Chen et al., 2020). In addition to serological methods, real-time fluorescent polymerase chain reaction (RT-PCR/qPCR) is widely utilized in clinical and laboratory settings due to its high specificity and sensitivity. Various qPCR assays have been developed targeting different genes, such as the cloned *PstI* gene fragment, major outer membrane protein gene (*ompA*), and 16S rRNA (Tondella et al., 2002; Hardick et al., 2004; Gullsby et al., 2008). While this method can reduce the risk of contamination between samples compared to traditional PCR or agarose gel electrophoresis (AGE), it requires specific instruments and involves longer reaction times. Among the three methods evaluated, serological detection and qPCR are currently the most widely used techniques for diagnosing *C. pneumoniae* infection. Serological methods detect specific antibodies against *C. pneumoniae* in patient serum specimens. In contrast, the qPCR method identifies *C. pneumoniae* directly in patient exudates or body fluids. Notably, the detection system established in this study aligns with the qPCR method in terms of antigen detection; thus, the primary comparison is made with the qPCR technique.

Recently, clustered regularly interspaced short palindromic repeats (CRISPR) and CRISPR-associated proteins (Cas) have become crucial tools in gene editing and nucleic acid detection (Swarts and Jinek, 2019). Cas12a, an endonuclease, exhibits both cis-cleavage and trans-cleavage activities. The process by which it performs cleavage is particularly noteworthy: in the CRISPR system, following the formation of a binary complex - “gRNA/Cas12a” between gRNA and the Cas12a protein, the gRNA specifically binds to one strand of the target DNA in a complementary manner. Concurrently, Cas12a recognizes the PAM site on the complementary strand of the target DNA and executes a cis-cleavage function near the PAM site. This action results in the establishment of a ternary complex known as “gRNA/Cas12a/target DNA”. Subsequently, Cas12a carries out a trans-cleavage function, cleaving any adjacent single-stranded DNA (Paul and Montoya, 2020).

**Figure 1:**
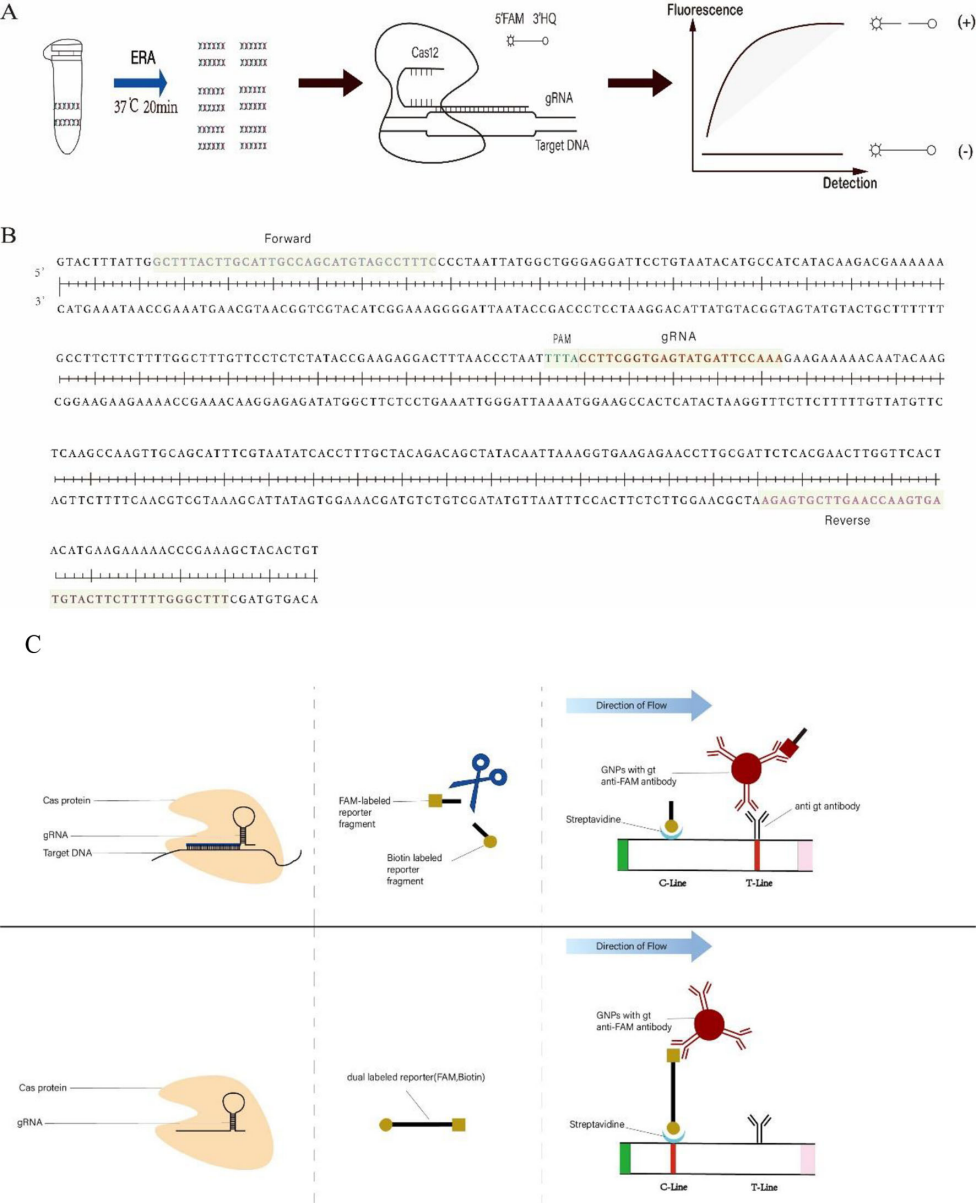
**(A)** ERA-CRISPR/Cas12a fluorescence system framework. The target sequence of *C*. *pneumoniae* DNA was amplified within 20 minutes using ERA. Subsequently, the Cas12a/gRNA complex identified the PAM site within the amplification product and cis-cleaved the target DNA. This action activated the trans-cleavage function of Cas12a, resulting in the disruption of reporters (F-Q). The resultant fluorescence signal was quantified using a fluorometer. **(B)** The sequence represents the target fragment of the *ompA* gene of *C*. *pneumoniae*. Forward and Reverse denote the primers used. PAM refers to the protospacer adjacent motif within the CRISPR array, followed by a sequence that binds to the gRNA. **(C)** ERA-CRISPR/Cas12a lateral flow system framework. The Cas12a/gRNA complex identified and cis-cleaved the target sequence and initiated trans-cleavage, resulting in the cleavage of a portion of the reporters (F-B) and fragmentation of the reporters (F-B). The uncleaved reporters bound colloidal gold ions and were fixed on the C-line. Meanwhile, the FAM-labeled end of the cleaved reporter bound colloidal gold ions and subsequently interacted with antibodies on the T-line in the direction of flow.

In these years, isothermal amplification technology has been increasingly applied in clinical settings. Techniques such as recombinase polymerase amplification (RPA) (Lobato and O’sullivan, 2018), recombinase aided amplification (RAA) (Mao et al., 2023), loop-mediated isothermal amplification (LAMP), rolling circle amplification (RCA), and enzymatic recombination amplification (ERA) have gained prominence (Zhao et al., 2015; Deng et al., 2022). By leveraging the dual functionalities of the CRISPR/Cas12a system combined with isothermal amplification technology, researchers have engineered fluorophores and fluorescence quenching groups at both ends of oligonucleotide sequences. When Cas12a, guided by gRNA, accurately performs cis-cleavage on the target double-stranded DNA (dsDNA), the modified oligonucleotide sequences undergo trans-cleavage. This trans-cleavage separates the fluorophores from the quenching groups, resulting in the emission of a detectable fluorescence signal, which can be quantified using a fluorometer (**Figure 1A**). Based on this innovative approach, detection systems have been successfully developed for pathogens such as *Trichomonas vaginalis*, *goose astrovirus*, and *Brucella* (Sullivan et al., 2019; Broughton et al., 2020; Dang et al., 2023). Additionally, alternative visual detection methods that do not require fluorometers have been introduced, such as colloidal gold lateral flow assay (Du et al., 2017; Li et al., 2021; Peng et al., 2024).

In this experiment, we combined the CRISPR/Cas12a system with enzymatic recombination amplification (ERA) technology to develop a two-step, dual-system detection method targeting the *ompA* gene of *C. pneumoniae*. The *ompA* gene can be amplified using the ERA kit in just 20 minutes at 37°C (**Figure 1A**). Following amplification, the CRISPR/Cas12a dual system can detect *C. pneumoniae* within 20-40 minutes (ERA-CRISPR/Cas12a fluorescence system) or 35 minutes (ERA-CRISPR/Cas12a lateral flow system). Notably, the results obtained from this method do not require specialized personnel for data analysis and can be observed under blue light or with the naked eye. This approach demonstrates good sensitivity and specificity, offering significant convenience for the detection of *C. pneumoniae* in grassroots public health units.

## Materials and methods

### Materials and reagents

The enzymatic recombinant amplification (ERA) kit for amplifying target DNA (KS101) was purchased from Suzhou Xianda Gene Technology Co., Ltd. (Suzhou, China). All oligonucleotides and recombinant plasmids were chemically synthesized by Sangon Biotech Co., Ltd. (Shanghai, China). The QIAamp DNA Mini Kit (No. 51306) for extracting bacterial DNA was obtained from QIAGEN GmbH (Germany). The Milenia GenLine HybriDetect kit (No. MGHD 1) for lateral flow system was purchased from Sansure Biotech Inc. (China). NEBuffer 2.1 is based on officially released reagent ingredients from New England BioLabs (No. B7202). The *Chlamydia trachomatis*, *C. pneumoniae*, and *Mycoplasma pneumoniae* nucleic acid detection kit (fluorescence PCR method) was acquired from Jiangsu Moeller Biotechnology Co., Ltd. for the detection of clinical specimens.

### Bacterial strains and clinical specimens

*C. pneumoniae* (*Cpn*, AR39), *Mycoplasma pneumoniae* (*Mp*, M129), *Chlamydia psittaci* (*Cps*, 6BC), *Chlamydia trachomatis* (*Ct*, serovar E), and *Staphylococcus aureus* (*SAU*, ATCC 25904) are deposited at the Institute of Pathogenic Biology, University of South China. *Klebsiella pneumoniae* (*Kpn*, ATCC 700603), *Mycobacterium tuberculosis* (*Mtb*, H37), *Streptococcus pneumoniae* (*Spn*, serovvar 3) was donated by the Department of Public Health Laboratory Sciences, College of Public Health, University of South China. DNA from these pathogens was extracted according to the QIAamp DNA Mini Kit instructions and stored in a refrigerator at -20°C. A total of 39 clinical respiratory pharyngeal swab specimens were collected from the First People’s Hospital of Chenzhou, Hunan Province, China. This collection includes 19 positive specimens and 20 negative specimens (where the *C. pneumoniae* infection test results were negative, but other upper respiratory pathogens may have tested positive).

### Primers and gRNA design

The *ompA* gene, the 16S rRNA gene, and the cloned *PstI* gene fragment are commonly used as target genes for qPCR methods (Villegas et al., 2010). It has been reported that the mature gRNA length of Cas12a ranges from 42 to 44 bp, and that Cas12a can recognize the PAM site with the 5’-TTTN-3’ sequence (Swarts and Jinek, 2019). Based on these principles, we designed and selected three gRNAs (**Table 1**) from the complete sequence of the *ompA* gene (> NC_002179.2: 1191670-1192248) (**Figure 1B**) in the National Center for Biotechnology Information (NCBI) database using https://www.zlab.bio/. Additionally, we designed four pairs of primers (**Table 2**) for the different gRNAs using Primer Premier 5 software. These primers exhibit high specificity in accordance with ERA primer design principles.

**Table 1:** Sequences of gRNAs for the dual system.

Name	Sequence (5'-3')
76gRNA	5'-UAAUUUCUACUAAGUGUAGAU CCUUCGGUGAGUAUGAUUCCAAA-3'
71gRNA	5'-UAAUUUCUACUAAGUGUAGAUGUAA UAUCACCUUUGCUACAGAC-3'
61gRNA	5'-UAAUUUCUACUAAGUGUAGAUCUACAGA CAGCUAUACAAUUAAA-3'

**Table 2:** Sequences of primers and probes for the dual system.

Name	Sequence (5'-3')
F_0_	5'-TTTACTTGCATTGCCAGCATGTAGCCTTTCC-3
R_0_	5'-TTTCTTCATGTAGTGAACCAAGTTCGTGAGA-3'
76F_2_	5'-GCTTTACTTGCATTGCCAGCATGTAGCCTTTC-3'
76R_2_	5'-TTTCTTCATGTAGTGAACCAAGTTCGTGAGA-3'
71F_2_	5'-CTAATTTTACCTTCGGTGAGTATGATTCCAA-3'
71R_2_	5'-ATGCAGCTCCACGCTCGTCAGTATGCCCTTC-3'
61F_2_	5'-CAATACAAGTCAAGCCAAGTTGCAGCATTTC-3'
61R_2_	5'-GTTATAGGATGCAGCTCCACGCTCGTCAGTA-3'
F/Q	5'-/6-FAM/TTATTATT/BHQ1/-3'
F/B	5'-/6-FAM/TTATTATT/Biotin/-3Y

### The standard enzymatic recombination amplification system

The QIAamp DNA Mini Kit amplification system consists of 20 µL of solubilizer, 2.5 µL of forward primer, 2.5 µL of reverse primer, 2-10 µL of template DNA, 21-13 µL of ddH_2_O, and 2 µL of activator, resulting in a total volume of 50 µL. The first four components were added sequentially to an EP tube, which was then transferred to the nucleic acid amplification EP tube. The reagents in the tube were re-suspended and mixed thoroughly. Following this, the ERA amplification product was placed in a 37°C water bath or incubator for 20 minutes after 2 µL of activator was added to the lid of the nucleic acid amplification EP tube.

### Establishment of the ERA-CRISPR/Cas12a fluorescence system

The total volume of this system is 50 µL. The detection methods are as follows: 0.12 µL of gRNA (5 µM), 0.1 µL of Cas12a (13 µM), 2 µL of 5× NEBuffer 2.1, and 7.6 µL of ddH_2_O were added to the synthesized premix solution and incubated in a 37°C water bath or incubator for 10 minutes. Subsequently, 1.5 µL of the F-Q probe (**Table 2**) (300 nM) was added to the premix to synthesize approximately 10 µL of the gRNA/Cas12a/F-Q complex. Finally, 10 µL of the gRNA/Cas12a/F-Q complex and 1 µL of the ERA amplification product were combined with 39 µL of 1× NEBuffer 2.1. After mixing, the LightCycler^®^ 96 real-time quantitative PCR was used to monitor the fluorescence intensity per minute.

### Establishment of the ERA-CRISPR/Cas12a lateral flow system

In the first step, 0.5 µL of gRNA (5 µM), 2.5 µL of Cas12a (2 µM), 1 µL of 5× NEBuffer 2.1, and 1 µL of ddH_2_O were added to form synthesized premix solution 1 and incubated in a 37°C water bath or incubator for 10 minutes. In step two, 5 µL of the above premixed solution 1, 10 µL of 5× NEBuffer 2.1, 2 µL of the F-B probe (**Table 2**) (100 µM), 5 µL of the ERA amplification product, and 28 µL of ddH_2_O were combined to synthesize 50 µL of premix solution 2, which was then incubated in a 37°C water bath or incubator for 15 to 20 minutes. In step three, 50 µL of premix solution 2 was mixed with 50 µL of HybriDetect Assay Buffer, the HybriDetect dipsticks were inserted into the mixture, and results were observed with the naked eye within 10 minutes.

### Sensitivity of the Cpn-ERA-CRISPR/Cas12a dual system

To evaluate the sensitivity of this dual system, we constructed the *ompA* gene sequence on the pUC57 vector as a standard product. The quantity of the initial DNA template was calculated using the following formula: Amount (copies/µL) = [DNA concentration (g/µL)/(plasmid length in base pairs × 660)] × 6.02 × 10²³. The initial DNA template was then serially diluted to concentrations of 10⁵, 10⁴, 10³, 10², 10¹, and 10^0^ copies/µL. A volume of 2 µL per concentration was used as the DNA template for ERA amplification. The lowest sensitivity was determined using the ERA-CRISPR/Cas12a fluorescence system and the ERA-CRISPR/Cas12a lateral flow system, respectively.

### Specificity of the Cpn-ERA-CRISPR/Cas12a dual system

The QIAamp DNA Mini Kit (No. 51306) was utilized to extract DNA from seven different standard bacterial strains. ERA amplification was performed on various extracted DNA templates, with ddH_2_O used as the negative control and *C. pneumoniae* as the positive control. The specificity of the system was assessed using the ERA-CRISPR/Cas12a fluorescence system and the ERA-CRISPR/Cas12a lateral flow system, respectively.

### Statistical analysis

Statistics and analysis of the data results were performed on GraphPad 9.0 software (GraphPad Software, San Diego, CA, USA). All data in this study were expressed as the mean with standard deviation (mean + SDs). One-way ANOVA was employed to examine differences among multiple groups. Differences were considered statistically significant when P<0.05.

## Result

### Feasibility analysis of Cpn-ERA-CRISPR/Cas12a dual system

**Figure 2:**
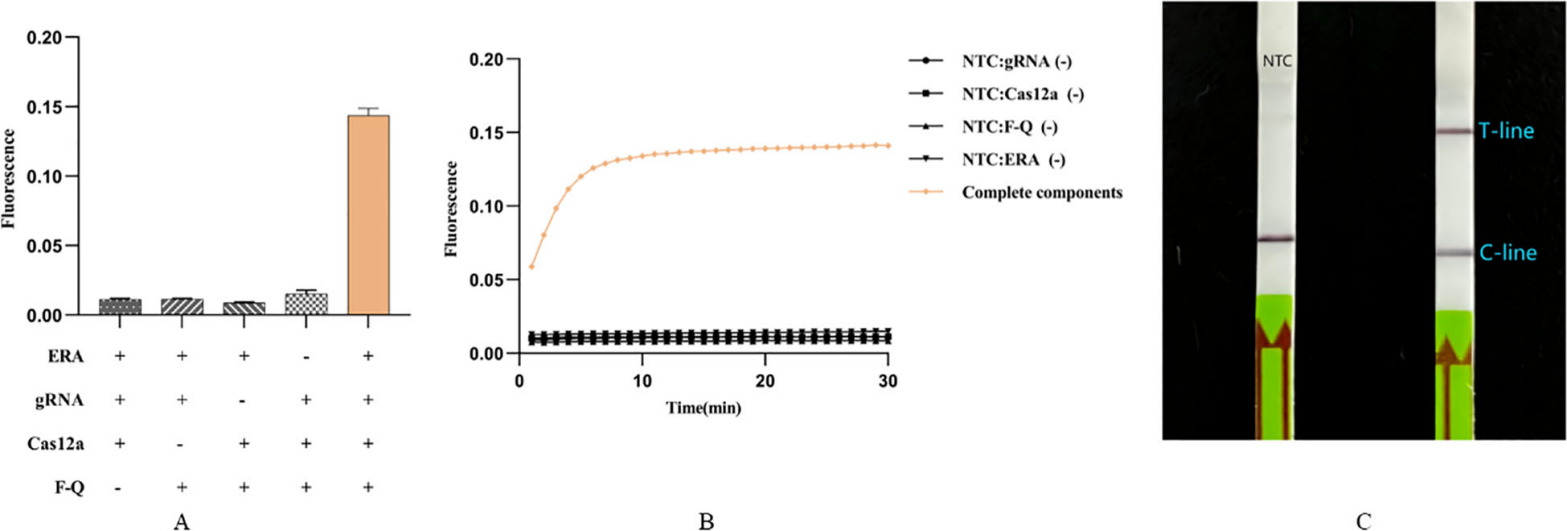
Feasibility analysis of Cpn-ERA-CRISPR/Cas12a dual system. **(A)** ERA-CRISPR/Cas12a fluorescence system: a strong fluorescence signal was detected after the reaction of ERA products and gRNA/Cas12a complexes with F-Q probes in a qPCR apparatus. **(B)** ERA-CRISPR/Cas12a fluorescence system: the fluorescence curve of ERA products and gRNA/Cas12a complexes reacting with F-Q probes changed with time within 30 minutes in a qPCR apparatu. **(C)** ERA-CRISPR/Cas12a lateral flow system. NTC: the amplification template for ERA is ddH_2_O. Error bars in panels represent the mean ± SD, where n = 3 replicates. The fluorescence values of all bars are the end values of the determination.

To reduce the time required for laboratory detection of *C. pneumoniae* infection, we combined the ERA method with the CRISPR/Cas12a system to develop a rapid, efficient, and sensitive detection system. The components of this system include the ERA amplification product, gRNA, Cas12a, and the F-Q probe, all of which are essential (**Figure 2A**). Initially, the fluorescence intensity increased gradually over time. After 10 minutes, the fluorescence intensity stabilized and entered the plateau phase, which was significantly different from that of the negative control (**Figure 2B**). The results demonstrated that the ERA-CRISPR/Cas12a fluorescence system can rapidly and accurately amplify the target gene at 37°C within 20 minutes. Detection results can be obtained at 37°C in 10 to 30 minutes, with the entire process taking only 40 to 60 minutes. It also demonstrated that the selected *ompA* gene can be cleaved by Cas12a.

Additionally, we established a visual detection method. After incubating premix solution 1 for 10 minutes and premix solution 2 for 15 minutes, results can be observed within 10 minutes of inserting the dipstick into the HybriDetect Assay Buffer. Including the ERA amplification time, the total process lasts only 55 minutes. Results showed that, compared to the negative control using ddH_2_O as the ERA amplification template, dipsticks containing intact components exhibited clear T-line and C-line, while the negative control displayed only C-line (**Figure 2C**).

### Optimization of Cpn-ERA-CRISPR/Cas12a dual system

#### Optimization of Primers and gRNA for ERA-CRISPR/Cas12a fluorescence system

**Figure 3:**
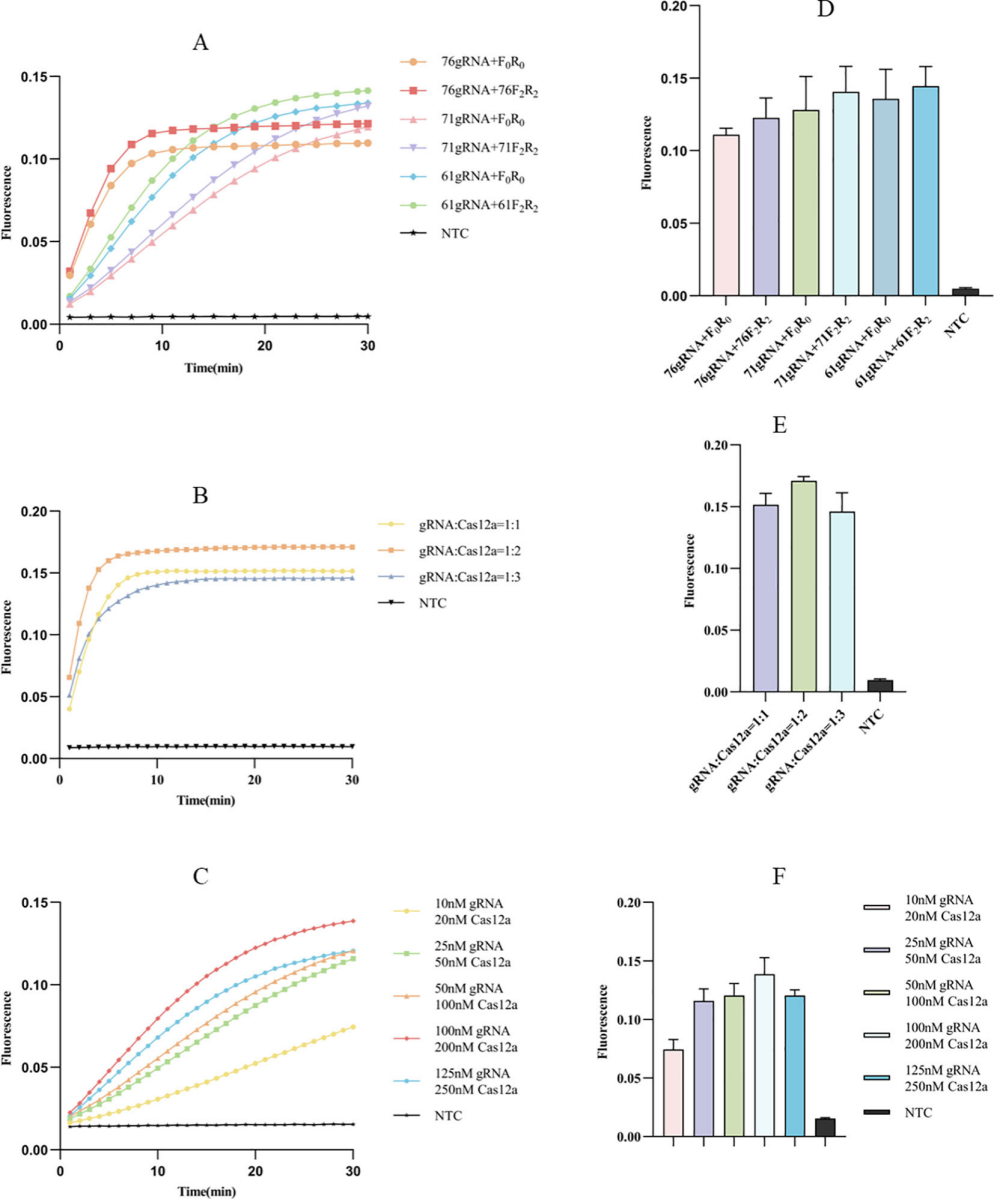
Optimization of fluorescence system. **(A, D)** Screening of primers and gRNAs: the fluorescence curve and intensity of different combinations (F_0_R_0_ is a universal pair of primers for 3 different gRNAs). **(B, E)** Ratio optimization of gRNA and Cas12a: the fluorescence curve and intensity of different ratio between gRNA and Cas12a. **(C, F)** Concentration optimization of gRNA and Cas12a: the fluorescence curve and intensity of different concentration for gRNA and Cas12a. NTC of all the above: the amplification template for ERA is ddH_2_O. Error bars in panels represent the mean ± SD, where n = 3 replicates. The fluorescence values of all bars are the end values of the determination.

We designed three gRNAs based on the *ompA* gene of *C. pneumoniae*: 76gRNA, 71gRNA, and 61gRNA, along with four corresponding primers: F_0_R_0_ (a universal primer pair for three different gRNAs), 76F_2_R_2_, 71F_2_R_2_, and 61F_2_R_2_. All three gRNAs can effectively guide Cas12a. Comparatively, the combination of 76gRNA and 76F_2_R_2_ reached the reaction plateau the fastest (**Figure 3A**). Although the fluorescence value of 61gRNA with its corresponding primers 61F_2_R_2_ was higher (**Figure 3D**), the time to reach the plateau was significantly longer than the combination of 76gRNA and 76F_2_R_2_ (**Figure 3A**). Therefore, in the final optimized ERA-CRISPR/Cas12a fluorescence system, we selected the combination of 76gRNA and 76F_2_R_2._

#### Optimization of Cas12a and gRNA concentration ratio for ERA-CRISPR/Cas12a fluorescence system

After selecting the optimal combination of gRNA and primer, we proceeded to investigate the optimal concentration ratio of gRNA to Cas12a. Observations indicated that at concentration ratios of 1:1 and 1:2, the reaction reached the plateau within 10 minutes (**Figure 3B**); however, the 1:2 ratio was faster and yielded a higher fluorescence value (**Figure 3E**). Additionally, we examined the optimal concentration of gRNA relative to Cas12a, finding that a gRNA concentration of 100 nM and a Cas12a concentration of 200 nM allowed the reaction to reach the plateau stage most quickly (**Figure 3C**), resulting in the highest fluorescence value (**Figure 3F**).

#### Optimization of probe concentration for ERA-CRISPR/Cas12a fluorescence system

**Figure 4:**
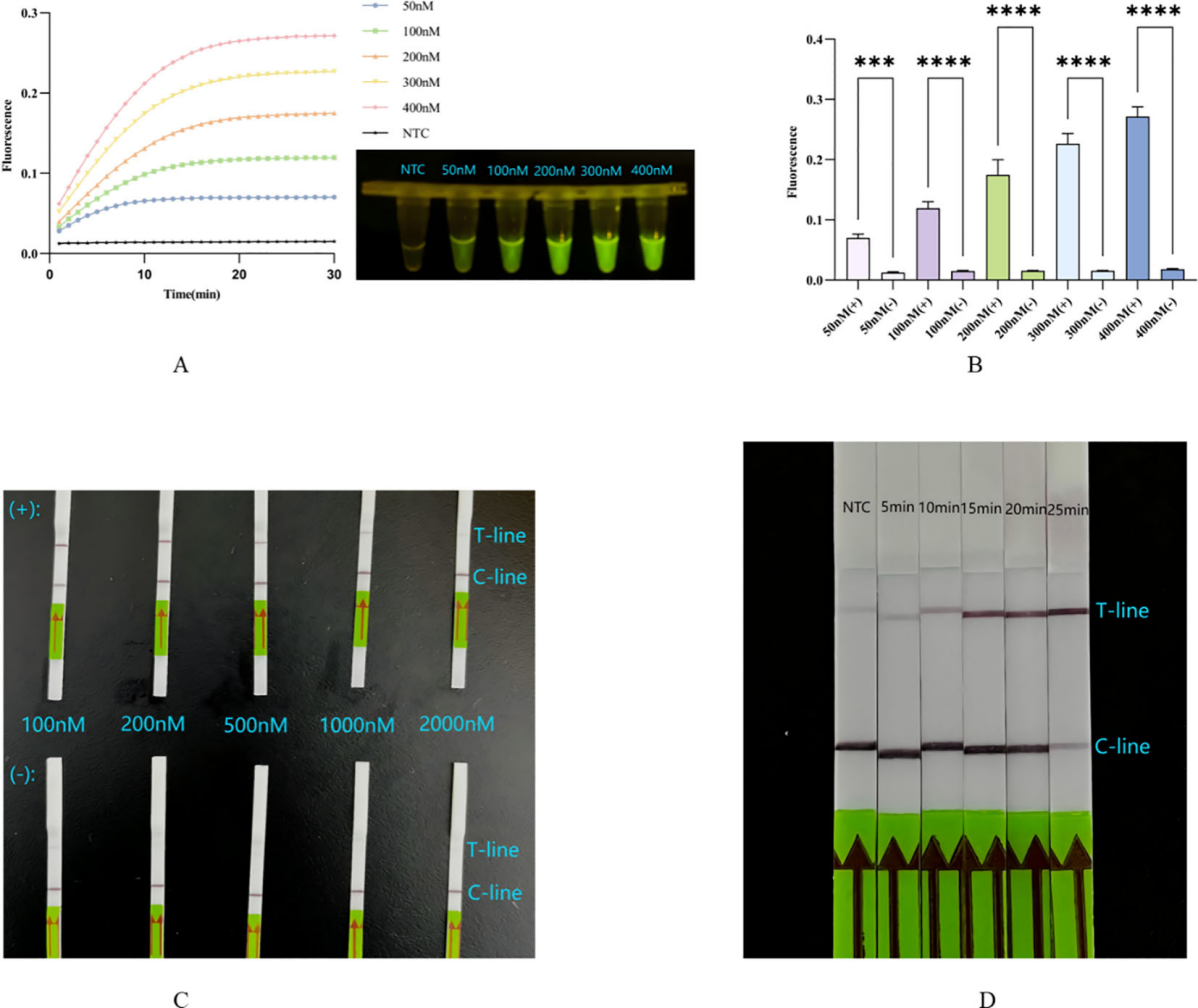
**(A, B)** Concentration optimization of probe for fluorescence system: the fluorescence curve and intensity of different concentrations for probes. ****, p<0.0001; ***, p<0.001. **(C)** Concentration optimization of probe for lateral flow system: in the top row are probes of different concentrations in the complete system, in the lower row are probes of different concentrations in the absence of Cas12a system. **(D)** Optimization of incubation time for lateral flow system. NTC: the amplification template for ERA is ddH_2_O. Error bars in panels represent the mean ± SD, where n = 3 replicates. The fluorescence values of all bars are the end values of the determination.

The concentration of the probe significantly affects the fluorescence value. The probe concentration gradient was diluted to 50 nM, 100 nM, 200 nM, 300 nM, and 400 nM. Calculations indicated that when the final probe concentration exceeds 50 nM, there is a significant difference between the probe and the corresponding negative control (**Figure 4B**), consistent with the blue light image (**Figure 4A**). Additionally, the probe concentration influences not only fluorescence intensity but also the reaction rate. We observed that the system responds more rapidly at higher probe concentrations (**Figure 4A**). Considering both the clarity of result interpretation and the experimental cost, we selected 300 nM as the optimal probe concentration.

#### Optimization of probe concentration for ERA-CRISPR/Cas12a lateral flow system

The unique working principle of the Milenia GenLine HybriDetect means that both excessively low and excessively high probe concentrations significantly impact result interpretation. The probe concentration gradient was diluted to 100 nM, 200 nM, 500 nM, 1000 nM, and 2000 nM. Results indicated that at probe concentrations of 100 nM and 200 nM, both the T-line and C-line were clearly visible. However, when the probe concentration reached ≥500 nM, the T-line became faint or even disappeared (**Figure 4C**). We inferred that this may be due to the number of uncleaved probes being much higher than that of cleaved probes, causing the uncleaved probes to bind a substantial amount of colloidal gold particles that were intended for the cleaved probes, resulting in the absence of color on the T-line even in the presence of a positive reaction, that is the famous hook effect (Sullivan et al., 2019; Broughton et al., 2020; Dang et al., 2023) (**Figure 1C**).

#### Optimization of incubation time for ERA-CRISPR/Cas12a lateral flow system

In comparison to the ERA-CRISPR/Cas12a fluorescence system, the progression of Cas12a in cleaving target DNA during the second step cannot be assessed due to the absence of instruments for monitoring real-time fluorescence values. Therefore, investigating the incubation time is essential for the lateral flow method. At the five-minute mark, no significant change was observed in the T-line compared to the negative control. However, when the cleavage time exceeded 15 minutes but was less than 20 minutes, clearer T and C lines became visible. Notably, at the 25-minute mark, the C-line appeared unclear (**Figure 4D**), possibly due to an extended reaction time leading to excessive consumption of the probe, but this result can also be interpreted as positive (Broughton et al., 2020).

### Specificity analysis of Cpn-ERA-CRISPR/Cas12a dual system

**Figure 5:**
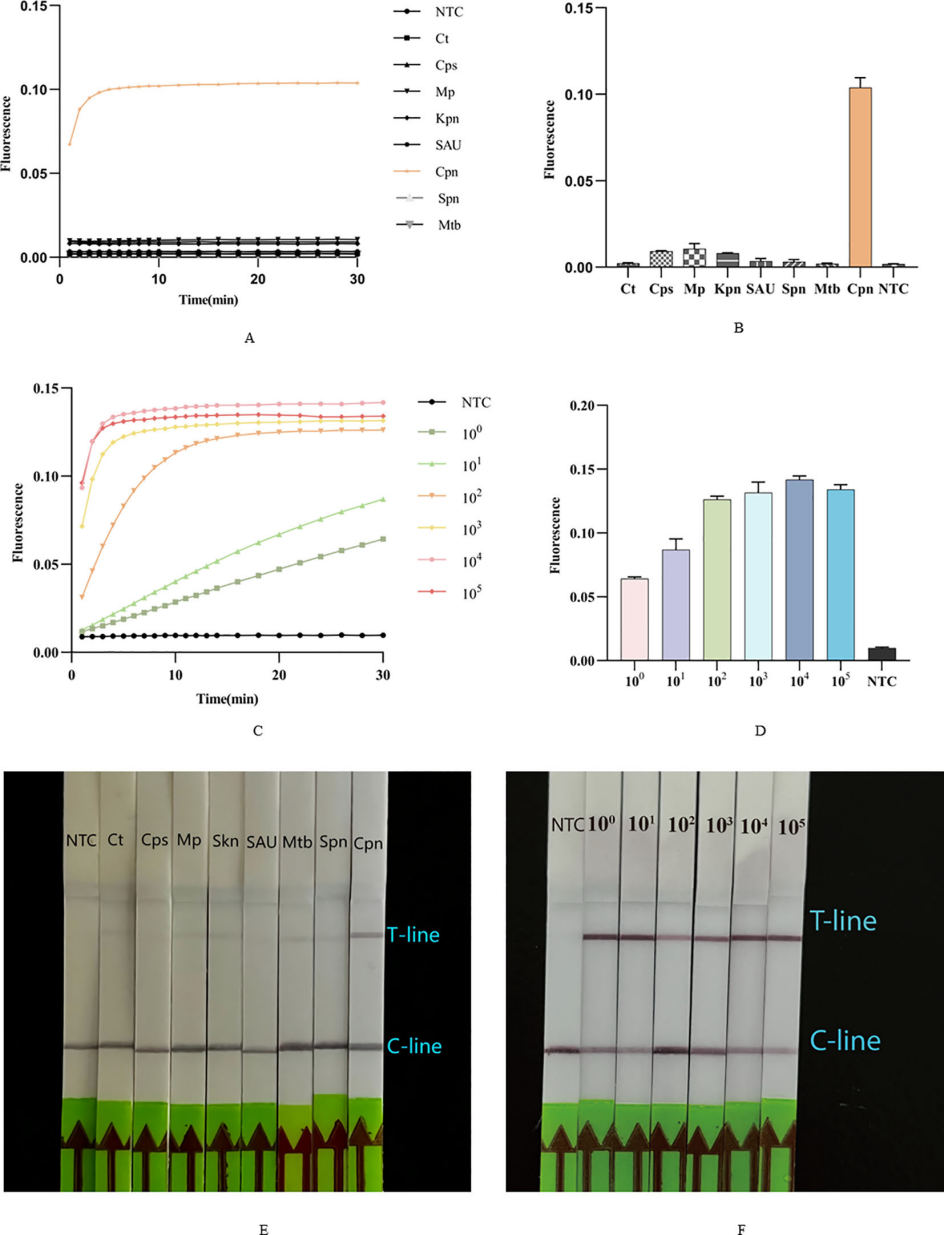
Sensitivity and specificity of Cpn-ERA-CRISPR/Cas12a Dual System. **(A, B)** The fluorescence curve and intensity of different bacteria. *Ct, Chlamydia trachomatis; Cps, Chlamydia psittaci; Mp, Mycoplasma pneumoniae; Kpn, Klebsiella pneumoniae; SAU, Staphylococcus aureus; Spn, Streptococcus pneumoniae; Mtb, Mycobacterium tuberculosis; Cpn, C. pneumoniae.* **(C, D)** The fluorescence curve and intensity of ERA-CRISPR/Cas12a fluorescence system at each dilution. **(E)** Specificity test of ERA-CRISPR/Cas12a lateral flow system. **(F)** Analytical sensitivity of ERA-CRISPR/Cas12a lateral flow system. NTC of all the above: the amplification template for ERA is ddH_2_O. Error bars in panels represent the mean ± SD, where n = 3 replicates. The fluorescence values of all bars are the end values of the determination.

We tested the specificity of this dual system using seven additional strains. The results indicated that, compared to these seven strains, only *C. pneumoniae* exhibited a statistically significant positive fluorescence curve (**Figure 5A**) and intensity (**Figure 5B**), while the others were negative. Consistent with the findings of the ERA-CRISPR/Cas12a fluorescence system, only the T-line of the lateral flow strip corresponding to *C. pneumoniae* was clearly visible, with no T-lines observed for the other strains (**Figure 5E**). These results suggest that the dual system demonstrates high specificity.

### Sensitivity analysis of Cpn-ERA-CRISPR/Cas12a dual system

Following construction of the standard *ompA* gene plasmid of *C. pneumoniae*, it was sequentially diluted to concentrations of 10^5^, 10^4^, 10^3^, 10^2^, 10^1^ and 10^0^ copies/µL. The results demonstrated that the ERA-CRISPR/Cas12a fluorescence system has a detection limit of 10^0^ copies/µ (**Figures 5C, D**). The ERA/CRISPR-LbCas12a lateral flow system also exhibited high sensitivity, as a clear T-line was observed at a template concentration of 10^0^ copies/µL (**Figure 5F**).

### Clinical sample validation

**Figure 6:**
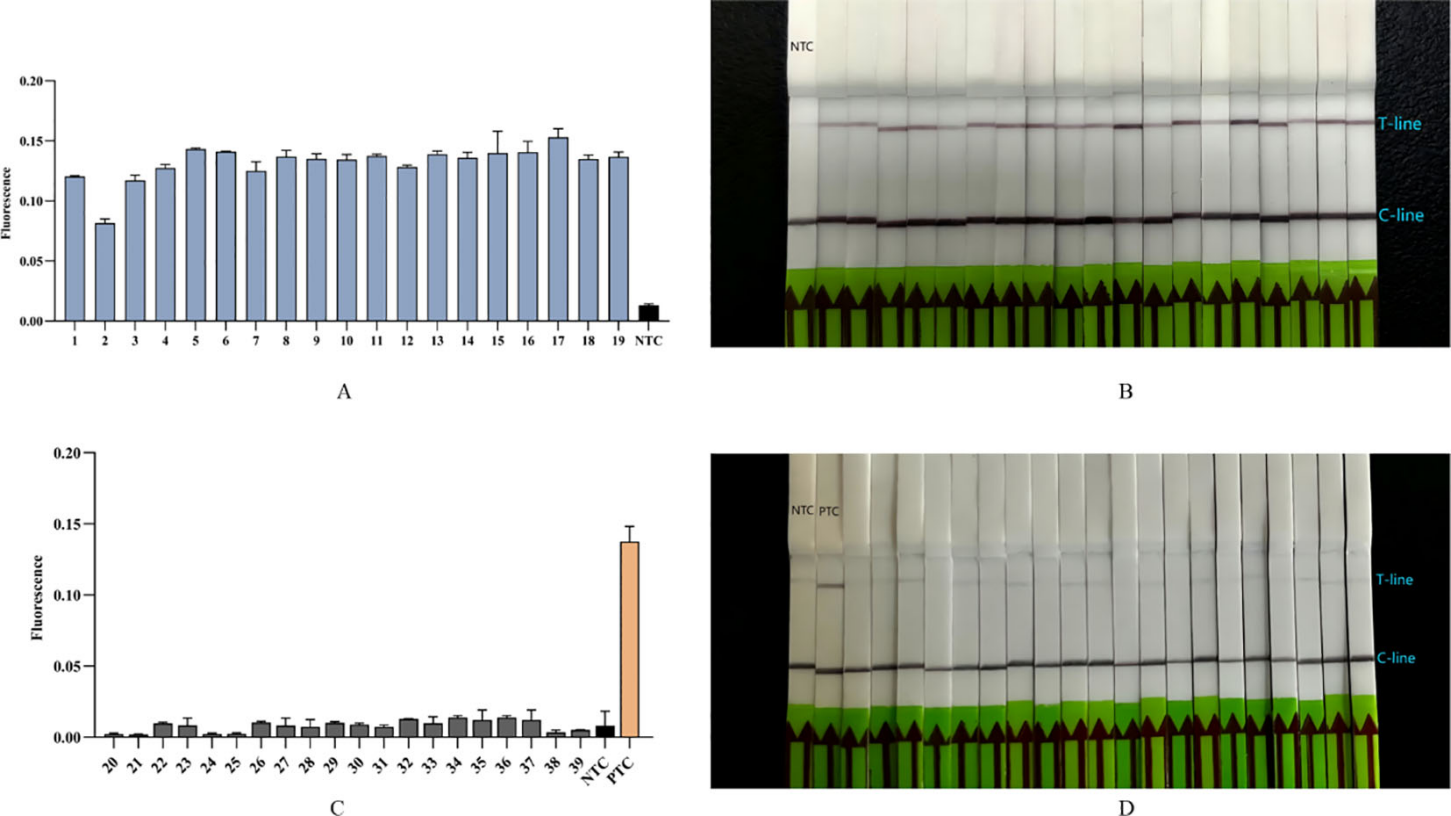
Detection of 39 clinical samples (19 positive specimens, 20 negative specimens) by Cpn-ERA-CRISPR/Cas12a dual system for *C. pneumoniae*. **(A)** The fluorescence intensity of 19 positive clinical specimens from ERA-CRISPR/Cas12a fluorescence system. **(B)** Results of 19 positive clinical specimens by ERA-CRISPR/Cas12a lateral flow system. **(C)** The fluorescence intensity of 20 negative clinical specimens from ERA-CRISPR/Cas12a fluorescence system. **(D)** Results of 20 negative clinical specimens by ERA-CRISPR/Cas12a lateral flow system. NTC of all the above: the amplification template for ERA is ddH_2_O. PTC: the amplification template for ERA is Standard plasmid. Error bars in panels represent the mean ± SD, where n = 3 replicates. The fluorescence values of all bars are the end values of the determination.

To assess the accuracy of the Cpn-ERA-CRISPR/Cas12a dual system in detecting complex clinical samples, including the detection rate in positive specimens and the specificity in negative specimens, we conducted parallel testing. Nucleic acid extracts were collected from 39 upper respiratory pharyngeal swabs, with qPCR results serving as the standard for comparison (19 specimens tested positive and 20 tested negative). The findings revealed that all 19 specimens identified as positive by qPCR (**Table 3**) also tested positive with the dual system (**Figures 6A, B**). Furthermore, the dual system did not yield any positive results among the 20 specimens classified as negative (**Figures 6C, D**).

**Table 3:** C_T_ of clinical samples.

Number	1	2	3	4	5	6	7	8	9	10
**C_T_**	33.20	34.35	32.00	32.60	26.63	26.94	31.50	32.24	29.39	29.42
Number	11	12	13	14	15	16	17	18	19	
**C_T_**	29.12	29.71	26.16	26.70	21.53	27.9	28.00	26.4	28.68	

CT<34.93 is a positive sample.

## Discussion

Surveillance of acute respiratory infection cases in China from 2009 to 2019 revealed that the infection rate of *C. pneumoniae* reached 1.6%, with an even higher rate of 2.6% among school-age children (Li et al., 2021). C*. pneumoniae* not only induces chronic coughs that are prone to relapse and difficult to treat but can also cause mixed infections with other bacteria (Du et al., 2017), posing a significant threat to public health. Currently, CRISPR/Cas systems have been used for rapid detection of a variety of bacteria and viruses, such as *Mycobacterium tuberculosis* (Peng et al., 2024), *White Spot Syndrome Virus in Shrimp* (Sullivan et al., 2019), *Burkholderia pseudomallei* (Deng et al., 2023). However, these methods have not yet been applied to the diagnosis of *C. pneumoniae* infections. Therefore, exploring this avenue represents a promising new research direction.

The OmpA protein, a common component of the outer membrane of Gram-negative bacteria with a molecular weight of approximately 28-36 kDa, is typically associated with pathogenic effects, including invasion, adhesion, and pro-inflammatory responses. It is frequently utilized as a target or candidate for vaccines aimed at the immune system (Confer and Ayalew, 2013). The *ompA* gene sequences of *C. pneumoniae* exhibit only 68% and 71% similarity to those of *Chlamydia trachomatis* and *Chlamydia psittaci*, respectively, making them valuable targets for qPCR and gene sequencing in bacterial identification (Kaltenboeck et al., 1993; Mahony et al., 2000; Kabeya et al., 2015). In this study, we designed ERA primers for the *ompA* gene, and our results demonstrated no cross-reaction with common pathogens, confirming the high specificity of the ERA-CRISPR/Cas12a dual system (**Figures 5A, B, E**).

PCR technology has been widely utilized in clinical specimen detection and gene sequencing analysis. However, whether employing real-time quantitative PCR (RT-qPCR), reverse transcription PCR (RT-PCR), nested PCR, or Touchdown Polymerase Chain Reaction, these methods require expensive instruments and specialized technicians with expertise to interpret experimental results, making the operations complex (Heid et al., 1996; Bachman, 2013; Green and Sambrook, 2018; Green and Sambrook, 2019). To address these challenges, isothermal nucleic acid amplification technology (INAAT) has emerged. Existing isothermal amplification techniques include enzyme-assisted INAAT, loop-mediated isothermal amplification (LAMP), rolling circle amplification (RCA), recombinase-aided amplification (RAA), recombinase polymerase amplification (RPA) (Mao et al., 2023; Wei et al., 2023)and so on. LAMP enables stable amplification within 30 minutes at temperatures of 60°C to 65°C using 4-6 primers (Soroka et al., 2021). It played a significant role during the 2019-NCOV public health crisis; however, LAMP has stringent requirements for primer design (Chaouch, 2021). RCA can amplify target genes after constructing a complete single-stranded DNA ring (Mohsen and Kool, 2016). Yutong Wang et al. successfully detected *S. aureus* using RCA technology combined with magnetic separation, achieving a low detection limit of 3.3 × 10³ CFU/mL (Wang et al., 2022). Nonetheless, RCA has higher requirements for DNA ligase. RPA can amplify 1-10 copies of the target gene within 20 minutes at 37°C to 42°C, demonstrating high sensitivity. It has been successfully applied in the detection of *Mycoplasma pneumoniae*, *Salmonella*, and *rabies virus* (Faye et al., 2021; Li et al., 2021; Li et al., 2022; Mao et al., 2023). Compared to LAMP, ERA primers are simpler to design and require fewer reaction reagents than RCA. Additionally, ERA represents an enhanced iteration of RPA, distinguishing itself by utilizing a different enzyme source and demonstrating increased efficiency through enzyme engineering (Wei et al., 2022; Liu et al., 2024). In this experiment, ERA technology allows the addition of a small amount (2-10 μL) of DNA template to obtain amplified products in a short time.

In this study, we combined enzymatic recombination amplification (ERA) with the CRISPR/Cas12a system. When Cas12a cis-cleaves the target gene, it can non-specifically trans-cleave the probe’s single strand. At this point, the fluorescence quenching agent at the 3’ end of the probe is separated from the fluorophore at the 5’ end, allowing for visualization of the CRISPR/Cas12a reaction. Regarding operational time, ERA amplification requires only 20 minutes. The fluorescence system reaches its plateau in 10 to 30 minutes, and results from the lateral flow system can be observed after 15 minutes of incubation. In contrast, the qPCR method typically takes 90 to 120 minutes to complete, highlighting the significant time-saving advantage of the dual system for healthcare workers and patients. In terms of reagent costs, the fluorescence system and lateral flow system each cost $3.50 and $7.50 per person, respectively, making them more economical than the $10 to $20 per person typically associated with commercial fluorescent qPCR kits in our region. Regarding equipment requirements, the lateral flow system requires only a vortex mixer, water bath or incubator. The fluorescence system, in addition to the above instruments, also necessitates a fluorometer. However, in resource-limited areas where access to an expensive qPCR fluorometer may be restricted, detection results can be interpreted directly under blue light. It is important to note that the results of the dual system are evaluated solely by the operator’s visual assessment, which demonstrates significant user-friendliness. Additionally, the experimental results indicate that both two systems can achieve a detection limit of 10^0^ copies/µL, exhibiting very high sensitivity. It is important to acknowledge that, compared to the qPCR method, the dual system, particularly the fluorescence system, has notable limitations. The results obtained from qPCR not only indicate the presence or absence of target DNA but also provide insights into the initial template concentration of target DNA through data analysis, offering more diagnostic information for clinicians. In the fluorescence system, we utilized ERA technology to amplify target DNA at 37°C. While the product manual indicates that this amplification process is exponential, the specific calculation method supporting this conclusion has not been officially disclosed. Additionally, during the final stage of the experiment, when the fluorometer monitors the fluorescence intensity of the sample per minute at the same temperature of 37°C—consistent with the ERA amplification conditions—the target gene may continue to amplify. Consequently, further investigation is needed to enhance the quantitative analysis of detection data from the dual system.

Finally, the results from 19 positive specimens and 20 negative specimens detected by qPCR served as the standard to evaluate the clinical accuracy of the Cpn-ERA-CRISPR/Cas12a dual system detection platform. The findings demonstrated that the detection rate for positive specimens was 100%, with no positive specimens identified among the negative specimens, indicating 100% concordance with the qPCR results. This suggests that the Cpn-ERA-CRISPR/Cas12a dual system detection platform exhibits high accuracy and specificity, making it a reliable method for diagnosing *C. pneumoniae* upper respiratory tract infections.

It is important to acknowledge that this experiment has several limitations. First, due to regional constraints, the number of specimens used for clinical validation is limited, which may affect the accuracy of the results. Therefore, investing in long-term efforts and fostering broader regional collaboration is essential. Second, the types of pathogen strains preserved in our laboratory are limited, and the specificity validation phase did not include respiratory viruses. This omission may impact the specificity validation results of the Cpn-ERA-CRISPR/Cas12a dual system. Third, common interferents in clinical samples, such as blood, pus, and antibiotics used for pneumonia treatment—including amoxicillin, roxithromycin, and azithromycin—may affect the detection performance of the dual system; further research is required to investigate these effects. Fourth, there is a risk of cross-contamination due to multiple openings and the transfer of target DNA amplification during the experiment. Future studies should consider optimizing operational procedures to mitigate this risk. Finally, the precise cleavage of target genes by Cas12a relies on the recognition of PAM sites, which influences the design of gRNA (Qu et al., 2024). The issues mentioned above cannot be resolved at present and will require further investigation in the future.

## Data Availability

The datasets presented in this study can be found in online repositories. The names of the repository/repositories and accession number(s) can be found in the article/supplementary material.
